# Serum Phosphorus Concentration and Coronary Artery Calcification in Subjects without Renal Dysfunction

**DOI:** 10.1371/journal.pone.0151007

**Published:** 2016-03-18

**Authors:** Kyung Sun Park, Jongha Park, Seong Hoon Choi, Seo Hee Ann, Gillian Balbir Singh, Eun-Seok Shin, Jong Soo Lee, Hyun Chul Chung

**Affiliations:** 1 Department of Internal Medicine, Ulsan University Hospital, University of Ulsan College of Medicine, Ulsan, South Korea; 2 Department of Radiology, Ulsan University Hospital, University of Ulsan College of Medicine, Ulsan, South Korea; Brigham and Women's Hospital, Harvard Medical School, UNITED STATES

## Abstract

Serum phosphorus (P) concentration is associated with coronary artery calcification (CAC) as well as cardiovascular events in patients with chronic kidney disease. It has been suggested that this relationship is extended to subjects without renal dysfunction, but further explorations in diverse races and regions are still needed. We performed a cross-sectional study of 2,509 Korean subjects (Far Eastern Asian) with an estimated glomerular filtration rate of ≥60 ml/min/1.73m^2^ and who underwent coronary computerized tomography. Serum P concentration was divided into pre-determined 4 categories: ≤3.2, 3.2< to ≤3.6, 3.6< to ≤4.0 and >4.0 mg/dL. Agatston score (AS), an index of CAC, was divided into 3 categories: 0, 0< to ≤100, and >100. A multinomial logit model (baseline outcome: AS = 0) was applied to estimate the odds ratio (OR) for each serum P category (reference: ≤3.2mg/dL). Mean age of subjects was 53.5±9.1 years and 36.9% were female. In the adjusted model, serum P concentration of 3.6< to ≤4.0 mg/dL and >4.0 mg/dL showed high ORs for AS of >100 [OR: 1.58, 95% confidence interval (CI): 1.04–2.40 and OR: 2.11, 95% CI: 1.34–3.32, respectively]. A unit (mg/dL) increase in serum P concentration was associated with 50% increase in risk of AS >100 (OR: 1.50, 95% CI: 1.16–1.94). A higher serum P concentration, even within a normal range, may be associated with a higher CAC in subjects with normal renal function.

## Introduction

Phosphorus (P) plays an essential role in various biological processes including energy metabolism, cellular signaling, nucleic acid synthesis and the stabilization of cell membranes. The serum P concentration is influenced by both uptake and excretion with homeostasis tightly regulated within a narrow range by the fibroblast growth factor-23 (FGF-23), 1,25-dihydroxy-cholecalciferol [1,25(OH)_2_D_3_], and parathyroid hormone (PTH). Elevated serum P concentration may link with vascular calcification pathophysiologically [1–5], and it has been accepted as an important risk factor for vascular calcification [6, 7] as well as cardiovascular (CV) outcomes [8–12] in patients with chronic kidney disease (CKD). An association between serum P concentration and vascular calcification has been extended in subjects with preserved renal function by previous studies [13–15], but more evidence from diverse population, especially beyond racial or regional difference, is needed to validate it.

In our previous study of 402 healthy Korean (Far Eastern Asian) subjects, we observed that coronary artery calcification (CAC) on coronary computerized tomography (CT) was positively correlated to serum P concentration measured 10 years earlier [16]. Although the study somewhat supported a temporality, the association needs to be further validated in a larger and different cohort. In this study, we conducted a cross-sectional study examining the association between serum P concentration and CAC using Agatston score (AS), as a predictor of future coronary events [17, 18], in a total of 2,509 healthy subjects without renal dysfunction.

## Methods

### Subjects

We retrospectively reviewed all subjects who underwent coronary CT for screening purpose at the General Health Promotion Center in Ulsan University Hospital, Ulsan, South Korea between January, 2009 and December, 2013. Included were subjects aged ≥18 years, those with an available serum P concentration at the time of CT, an estimated glomerular filtration rate (eGFR) was ≥60 ml/min/1.73 m^2^ and a negative dipstick test for albuminuria. eGFR was calculated with the Modification of Diet in Renal Disease equation: eGFR = 186 × (serum creatinine)^-1.154^ × age^-0.203^ × [0.742 if female] [19, 20]. Excluded were those with a previous history of overt vascular events which were defined as: 1) coronary artery disease requiring intervention or medical treatment, 2) cerebral infarction or hemorrhage, and 3) atherosclerotic peripheral artery disease. This study was approved by the Institutional Review Board (IRB) of ethics committees at Ulsan University Hospital (IRB No. 2015-05-015). Subjects’ information was anonymized and de-identified prior to analysis. Given anonymity of the subjects studied, and the nonintrusive nature of the research, requirement for written informed consent was exempted by the IRB.

### Data Collection

Data was obtained using the Electronic Medical Recording system of Ulsan University Hospital. Serum P concentration was the main exposure. Covariates included age, sex, diabetes mellitus (DM), hypertension (HTN), body mass index (BMI), systolic blood pressure (SBP), corrected serum calcium, hemoglobin A1c (HbA1c), albumin, high-density lipoprotein (HDL) cholesterol, and low-density lipoprotein (LDL) cholesterol. Each participant completed a self-administered questionnaire which assessed medical history of HTN, DM, CV diseases, or current medications. DM was defined as fasting serum glucose ≥126 mg/dL, HbA1c ≥6.5% or the use of blood glucose lowering agents [21]. HTN was defined as a SBP ≥140 mmHg, diastolic BP ≥90 mmHg or current use of antihypertensive medication [22]. Blood sampling was done at morning after at least 8-hour fasting duration. Serum P concentration was measured using an automated clinical chemistry analyzer (Modular P analyzers; Roche Diagnostics, Tokyo, Japan). All reagent, calibrator, and control were used according to the manufacturer’s recommendation. The coefficient of variation for serum P determination was <1.8% and <1.9% for low and high level quality control specimens, respectively. Corrected serum calcium concentration was calculated as: corrected serum calcium (mg/dL) = total serum calcium (mg/dL) + 0.8 × [4.0—serum albumin (g/dL)]. CAC, the main outcome, was quantified as the AS on coronary CT [23–25]. All CT imaging were performed using a 3-mm thickness 256-slice CT scanner (Brilliance iCT, Philips Medical Systems, The Netherlands) with prospective ECG triggering. Scan parameters were as follows: tube voltage, 120 kVp; tube current, 100 mAs; 220 mm field of view; rotation time, 0.27 s/rotation; reconstructed slice thickness, 0.8-mm; signal acquisition at 80% of the R-R interval. There were no gaps between the slices. CAC was measured using the Extended Brilliance Workspace workstation (Philips Medical Systems, The Netherlands) after reconstruction. CAC was automatically displayed in color by calcium scoring software. Attenuation above the threshold of 130 HU was automatically marked as potential calcifications, and an experienced radiologist identified CAC by noise detection with manual placement at regions of interest showing CAC. Total AS was calculated by adding all values of the left main, left anterior descending, left circumflex and right coronary artery.

### Statistical Analysis

Data were expressed as mean ± standard deviation for continuous variables and as percentage (%) for discrete variables. Serum P concentration was divided into pre-defined 4 categories: ≤3.2, 3.2< to ≤3.6, 3.6< to ≤4.0 and >4.0 mg/dL. We set 3.6 mg/dL as midpoint based on the mean concentration found from our previous research but the range of each group, 0.4 mg/dL was chosen arbitrarily [16]. AS was divided into 3 categories: 0, 0< to ≤100, and >100. Multinomial logit model (baseline outcome: AS = 0) was applied to estimate the odds ratio (OR) for each serum P category (reference: ≤3.2mg/dL). Three levels of stepwise adjustments were done: 1) unadjusted model, 2) case-mix adjusted model including age, sex, DM and HTN, 3) fully adjusted model including all of covariates in the model 2 plus BMI, SBP, corrected serum calcium, albumin, HbA1c, LDL and HDL cholesterol. In data for covariates, there were no missing values for age, sex, DM, HTN and BMI. Percentages of missing in SBP, corrected serum calcium, albumin, HbA1c, LDL and HDL cholesterol were 1.2%, 0.2%, 0.2%, 12.4%, 5.9% and 5.8%, respectively. Missing values were imputed using the mean of the existing values according to serum P categories. We undertook sensitivity analyses separately without imputing missing data, and compared with main results. Statistical analyses were done using STATA MP Version 12. All statistical tests were two-sided and *P <*0.05 was considered significant.

## Results

### Baseline characteristics

A total of 3,523 subjects underwent coronary CT during the screening period. 60 subjects had two coronary CTs performed with a 2-year interval between each scan, but only the first CT data was included in the analysis. 954 subjects were excluded for the following reasons: 468 subjects had missing serum P concentration; 34 subjects had previous history of overt vascular events; and 452 subjects had an eGFR below 60 ml/min/1.73m^2^ and/or albuminuria on urine dipstick. Thus, remaining were 2,509 subjects were included for analysis.

The mean age of the subjects was 53.5 ± 9.1 years and 36.9% were female. All subjects were Far Eastern Asian by racial origin. 9.7% of subjects had DM and 21.4% had hypertension. The prevalence of DM was similar to that observed in the general population in South Korea (male 12.8%, female 9.1%), but prevalence of HTN was lower than the reported national figures (male 32.4%, female 22.2%) [26]. Mean serum creatinine concentration was 0.9 ± 0.2 mg/dL and mean eGFR was 79 ± 12 mL/min/1.73 m^2^. Mean serum P concentration was 3.7 ± 0.6 mg/dL (normal range in adults: 2.5–4.5 mg/dL).

Baseline characteristics according to serum P categories are summarized in Table 1. Patients’ age was slightly older and the proportion of women gradually increased in higher serum P categories. Mean serum P concentration was higher in women than in men (3.8 ± 0.5 mg/dL *vs*. 3.6 ± 0.5 mg/dL, *p* < 0.001). The prevalence of DM was similar among the categories, but HTN was observed less frequently in serum P concentration of 3.2< to ≤3.6 mg/dL. BMI significantly decreased along with increase in serum P concentration. The difference, however, was not large and correlation coefficient was small (*r* = -0.06, *p* = 0.003). Systolic BP was slightly lower in high serum P categories. Corrected serum calcium concentration increased along with serum P concentration, and showed a weak correlation (*r* = 0.10, *p* < 0.001). Serum albumin concentration did not differ in clinical aspect among serum P categories although mean values were different statistically (*p* = 0.02). LDL cholesterol concentration was lower in serum P concentration of ≤3.2 mg/dL, whereas HDL cholesterol was higher in that of >4.0 mg/dL.

**Table 1:** Baseline characteristics according to serum phosphorus concentrations.

	Total	Serum phosphorus (mg/dL)				p
		≤3.2	3.2< to ≤3.6	3.6< to ≤4.0	>4.0	
Number	2509	531	720	692	560	
Age, years (SD)	53.5 (9.1)	53.1 (9.2)	53.1 (8.9)	53.8 (9.2)	54.0 (9.2)	0.001
Sex, female %	36.8	23.8	27.3	38.3	59.8	< 0.001
DM, %	9.7	10.9	10.5	8.8	8.8	0.460
HTN, %	21.4	25.9	18.8	20.4	22.0	0.020
BMI, kg/m^2^ (SD)	24.2 (2.8)	24.5 (3.0)	24.2 (2.6)	24.1 (2.8)	24.0 (3.0)	0.001
SBP, mmHg (SD)	124 (16)	126 (17)	124 (15)	123 (16)	122 (16)	< 0.001
Corrected Ca, mg/dL (SD)	8.9 (0.5)	8.8 (0.4)	8.9 (0.5)	8.9 (0.5)	9.0 (0.5)	< 0.001
Albumin, g/dL (SD)	4.3 (0.4)	4.3 (0.4)	4.3 (0.4)	4.3 (0.4)	4.3 (0.4)	0.020
HbA1c, % (SD)	5.8 (0.8)	5.8 (0.8)	5.8 (0.9)	5.8 (0.8)	5.8 (0.8)	0.130
LDL cholesterol, mg/dL (SD)	119 (34)	115 (33)	121 (32)	119 (34)	122 (36)	0.020
HDL cholesterol, mg/dL (SD)	51 (13)	50 (13)	50 (13)	51 (13)	53 (13)	< 0.001
Agatston score group, %						0.520
= 0	63.3	62.1	64.9	61.9	63.9	
>0 to 100	24.5	26.6	24.0	25.0	22.5	
>100	12.2	11.3	11.1	13.1	13.6	

Abbreviations: SD; standard deviation, DM; diabetes mellitus, HTN; hypertension, BMI; body mass index, SBP; systolic blood pressure, Ca; calcium, HbA1c; hemoglobin A1c, LDL; low-density lipoprotein, HDL; high-density lipoprotein.

### Serum phosphorus concentration and coronary artery calcification

Among the 2,509 subjects, 1,587 subjects (63.2%) had an AS of 0, 615 (24.5%) had AS of 0< to ≤100, and 307 (12.2%) had AS of >100. As shown in Table 1, the proportion of 3 AS levels did not differ significantly among the serum P categories, but the prevalence of AS of >100 tended to be slightly higher in the serum P concentrations of 3.6< to ≤4.0 and >4.0 mg/dL.

**Fig 1:**
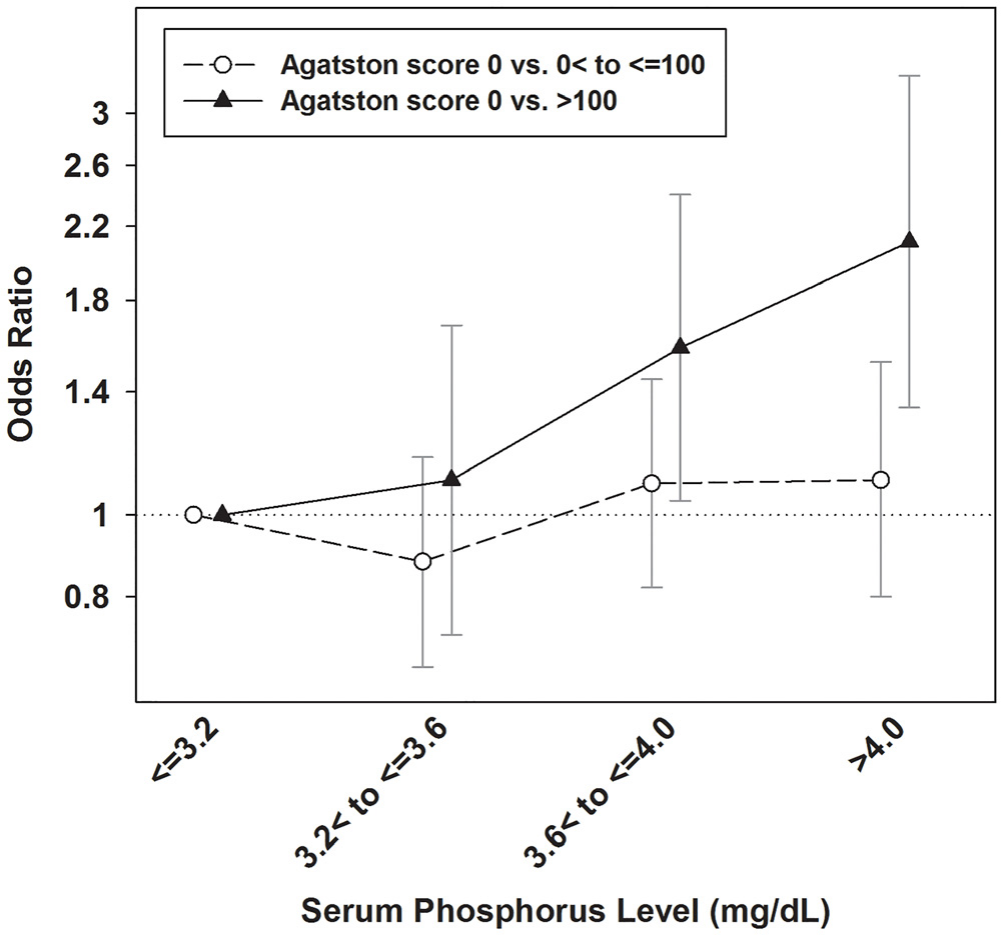
Adjusted odds ratios of two Agatston score categories by serum phosphorus concentrations. The model was adjusted for age, sex, diabetes, hypertension, body mass index, systolic blood pressure, corrected serum calcium, albumin, hemoglobin A1c, low-density lipoprotein cholesterol and high-density lipoprotein cholesterol. Y-axis used log scale.

In the unadjusted model, when modeling both AS of 0 *vs*. 0< to ≤100 and 0 *vs*. >100, the ORs for high AS were not significantly increased in serum P concentrations of 3.2< to ≤3.6 mg/dL, 3.6< to ≤4.0 mg/dL and >4.0 mg/dL compared with that of ≤3.2 mg/dL (Table 2). However, in the case-mix adjusted model, the ORs for subjects to have AS of >100 was significantly higher in serum P concentration of 3.6< to ≤4.0 mg and >4.0 mg/dL [OR: 1.52, 95% confidence interval (CI): 1.01–2.30) and OR: 2.01, 95% CI: 1.28–3.13]. Similar result was observed in the fully adjusted model. Serum P concentration was not significantly related with risk for AS of 0< to ≤100. However, serum P concentrations of 3.6< to ≤4.0 mg/dL and >4.0 mg/dL was associated with 58% and 111% higher risks of AS over 100 [OR: 1.58, 95% CI: 1.04–2.40 and OR: 2.11, 95%: 1.34–3.32]. ORs estimated in the fully adjusted model are presented graphically in Fig 1.

**Table 2:** Odds ratios for Agatston score categories according to serum phosphorus concentrations.

	Agatston score			
	0< to ≤100		>100	
Phosphorus (mg/dL)	OR (95% CI)*	*P*	OR (95% CI)*	*p*
Unadjusted				
≤3.2 (reference)	1.00		1.00	
3.2< to ≤3.6	0.86 (0.66–1.12)	0.27	0.94 (0.66–1.36)	0.75
3.6< to ≤4.0	0.94 (0.72–1.22)	0.66	1.17 (0.82–1.67)	0.39
>4.0	0.82 (0.62–1.09)	0.17	1.17 (0.81–1.70)	0.40
Case-mix adjusted^†^				
≤3.2 (reference)	1.00		1.00	
3.2< to ≤3.6	0.90 (0.68–1.20)	0.48	1.09 (0.72–1.65)	0.69
3.6< to ≤4.0	1.09 (0.82–1.45)	0.55	**1.52 (1.01–2.30)**	**0.047**
>4.0	1.14 (0.83–1.56)	0.41	**2.01 (1.28–3.13)**	**0.002**
Fully adjusted^‡^				
≤3.2 (reference)	1.00		1.00	
3.2< to ≤3.6	0.88 (0.66–1.17)	0.39	1.10 (0.72–1.68)	0.45
3.6< to ≤4.0	1.09 (0.82–1.45)	0.57	**1.58 (1.04–2.40)**	**0.03**
>4.0	1.10 (0.80–1.52)	0.55	**2.11 (1.34–3.32)**	**0.001**

* The multinomial logit model (baseline outcome category: Agatston score = 0) was used to calculate the odds ratio (OR) and 95% confidence interval (CI).

^†^ The case-mix adjusted model included age, sex, diabetes and hypertension.

^‡^ The fully adjusted model included all of covariates in the case-mix adjusted model and body mass index, systolic blood pressure, corrected serum calcium, albumin, hemoglobin A1c, LDL cholesterol and HDL cholesterol additionally.

When treating serum P concentration as a continuous variable, a unit (mg/dL) increase in serum P concentration was associated with 18% increase in risk for AS of 0< to ≤100 (OR: 1.18, 95% CI: 0.97–1.42) and 50% increase in risk for AS of >100 (OR: 1.50, 95% CI: 1.16–1.94) in the fully adjusted model.

### Covariates and coronary artery calcification

Among the covariates included in this analysis, old age, male gender, HTN, high SBP, high HbA1c, high LDL and low HDL cholesterol were independently associated with CAC, as expected. BMI, corrected serum calcium and albumin concentrations, however, were not (Table 3).

**Table 3:** Odds ratios for Agatston score categories according to covariates.

	Agatston score			
	0< to ≤100		>100	
Covariates	OR (95% CI)*	*P*	OR (95% CI)*	*p*
Age (per year)	**1.08 (1.07–1.96)**	**<0.001**	**1.17 (1.15–1.20)**	**<0.001**
Male	**2.64 (2.08–3.35)**	**<0.001**	**4.26 (3.27–6.61)**	**<0.001**
DM	1.25 (0.79–1.96)	0.34	1.66 (0.97–2.83)	0.06
HTN	**1.70 (1.29–2.25)**	**<0.001**	**1.56 (1.09–2.24)**	**0.015**
BMI (kg/m^2^)	1.03 (0.99–1.07)	0.17	1.04 (0.99–1.10)	0.14
SBP (10 mmHg)	1.03 (0.96–1.11)	0.38	**1.17 (1.06–1.29)**	**0.001**
Corrected Ca (mg/dL)	1.13 (0.91–1.41)	0.27	1.30 (0.96–1.77)	0.09
Albumin (g/dL)	0.81 (0.61–1.08)	0.15	0.87 (0.59–1.29)	0.48
HbA1c (%)	**1.30 (1.08–1.56)**	**0.005**	**1.37 (1.11–1.70)**	**0.004**
LDL (10 mg/dL)	**1.05 (1.02–1.08)**	**0.03**	0.96 (0.92–1.01)	0.12
HDL (10 mg/dL)	**0.91 (0.83–0.99)**	**0.026**	**0.84 (0.74–0.95)**	**0.007**

Abbreviations: DM, diabetes mellitus; HTN, hypertension; BMI, body mass index; SBP, systolic blood pressure; Ca, calcium; HbA1c; hemoglobin A1c, LDL, low-density lipoprotein cholesterol; HDL, high-density lipoprotein cholesterol.

*The multinomial logit model (baseline outcome category: Agatston score = 0) was used to calculate the odds ratio (OR) and 95% confidence interval (CI).

### Sensitivity analyses

Separate analyses without imputing missing covariates also showed comparable results with those after imputation (Table 4). After dividing AS into dichotomized categories (AS ≤100 *vs*. >100), adjusted ORs were estimated by serum P categories with logistic regression. ORs for AS of >100 were 1.53 (95% CI: 1.03–2.28) and 2.06 (95% CI: 1.34–3.17) in serum P concentration of 3.6< to ≤4.0 mg/dL and >4.0 mg/dL, respectively. A unit increase in serum P concentration showed OR of 1.40 (95% CI: 1.10–1.79) for AS of >100.

**Table 4:** Number of subjects and odds ratios for Agatston score categories according to serum phosphorus concentrations in fully adjusted models^†^ without imputation of missing covariates (n = 2147).

**No of subject**	**Agatston score**			
**Phosphorus (mg/dL)**	**total**	**0**	**0< to** ≤**100**	**>100**
≤3.2 (reference)	432	274 (63.4%)	112 (25.9%)	46 (10.6%)
3.2< to ≤3.6	638	420 (65.8%)	145 (22.7%)	73 (11.4%)
3.6< to ≤4.0	596	377 (63.3%)	146 (24.5%)	73 (12.2%)
>4.0	481	315 (65.5%)	105 (21.8%)	59 (12.3%)
	**Agatston score**			
	**0< to** ≤**100**		**>100**	
**Phosphorus (mg/dL)**	**OR (95% CI)**	***P***	**OR (95% CI)**	***P***
≤3.2 (reference)	1.00		1.00	
3.2< to ≤3.6	0.86 (0.63–1.17)	0.35	1.23 (0.78–1.96)	0.38
3.6< to ≤4.0	1.08 (0.80–1.48)	0.62	1.53 (0.95–2.44)	0.08
>4.0	1.11 (0.78–1.57)	0.56	**2.04 (1.22–3.41)**	**0.006**

^†^ The multinomial logit model (baseline outcome category: Agatston score = 0) was used to calculate the odds ratio (OR) and 95% confidence interval (CI). The model included age, sex, DM, HTN, body mass index, systolic blood pressure, corrected serum calcium, albumin, HbA1c, LDL cholesterol and HDL cholesterol.

## Discussion

This study showed that a higher serum P concentration was significantly associated with CAC in Far Eastern Asian subjects with preserved renal function. The association was observed even when serum P was within the normal range.

In CKD, body P is retained and serum P concentration increases because of decrease in overall functioning nephrons [27]. Hyperphosphatemia has emerged as an important non-traditional risk factor for CV disease and mortality in CKD patients [8–12]. These findings have been extended to subjects with preserved renal function [28, 29]. Foley *et al*. observed that a higher serum P concentration was associated with greater likelihood of higher CAC score categories in 3,015 young adults with normal renal function [13]. Cancela *et al*. found that, in 209 patients undergoing elective coronary angiography (creatinine clearance >60 ml/min/1.73m^2^), each 0.1-mg/dL rise in serum P concentration was associated with a 9.2% higher odds of having AS of >10 [14]. These two studies, however, observed limited populations: young white and African-American adults, and Brazilian (white and non-white) patients undergoing elective coronary angiography. It is worthy to explore the association in racially and regionally different populations. In our previous study of 402 Korean subjects with eGFR >60 ml/min/1.73m^2^, we estimated an OR of 3.89 (95% CI: 1.43–10.63) for an AS of >100 in those with serum P of 3.6< to ≤3.9 mg/dL and an OR of 3.17 (95% CI: 1.19–8.41) in those with serum P concentration of >3.9 mg/dL compared to those with serum P concentration of <3.3 mg/dL [16]. In this study with different and larger number of subjects, we validated a serum P—CAC association in Asian peoples again. Subjects with serum P concentration of 3.6< to ≤4.0 mg/dL and >4.0 mg/dL showed 58% and 111% higher risks for having AS of >100 compared to serum P concentration of ≤3.2 mg/dL (OR: 1.58, 95% CI: 1.04–2.40 and OR: 2.11, 95% CI: 1.34–3.32, respectively). The ORs were smaller than those of our previous report, and 95% CI were narrower. It might be due to large sample size in part. A recent report from other large Korean cohort without CKD (n = 23,652) was comparable with our observation, although the subjects tended to be younger (mean age: 40.8±7.3 years) and male dominantly (83.5%). Kwak *et al*. found that the prevalence ratio (95% CI) comparing the highest quartile of serum P concentration (≥3.9 mg/dL) to the lowest quartile (<3.4 mg/dL) were 1.70 (1.50–1.93) for AS of 1–100 and 1.93 (1.43–2.61) for AS of >100 [15].

Although a full biological understanding of these findings has not been reached yet, the relation of serum P concentration with CAC may be important concern in the view of public health. In above-mentioned studies, even a high normal serum P concentration was associated with risk of CAC. Potential interventions that can decrease P burden or serum P concentration already exist [30, 31]. Our findings suggests that clinical trials to high-risk individuals in general population has the potential to improve public health [32]. We used AS for scoring the amount of CAC. The AS is the product of the within-slice CAC plaque area and a plaque-specific density factor which reflects increasing categories of Hounsfield unit, summed for all cardiac CT slices. Thus, the AS is weighted upward for greater CAC density. Criqui *et al*. reported that CAC volume was positively associated with coronary heart disease and CV disease risk, but at any level of CAC volume, CAC density was inversely associated with the risks [33]. In these lines, serum P concentration needs to be examined against CAC density, which may allow us to better assess the risk.

This study has several limitations. Firstly, there was no data collected on serum FGF-23, intact PTH, and calcitriol concentrations, which are key regulatory hormones for serum P concentration. However, subjects with eGFR >60 ml/min/1.73m^2^ are unlikely to have abnormal intact PTH and calcitriol levels. The potential confounding from the absence of this intact PTH and calcitriol data is minimal but cannot be completely excluded. The possible association between serum FGF-23 concentration and CAC has been suggested [34, 35] but a recent report showed that baseline plasma FGF-23 was not associated with the prevalence or severity of CAC. And a higher serum P level was associated with CAC, even after adjustment for FGF-23 level [36]. In subject without renal insufficiency, serum FGF-23 was not related with CAC and did not affect serum P—CAC association [14]. Second, dietary intake of calcium and P was not quantified and incorporated. The reason for a higher serum P concentration could not be elucidated from this study. Third, it was possible that co-morbid conditions such as mild pre-existing CV disease or other chronic illness were not identified because we included asymptomatic individuals who underwent a general health examination. Smoking status and use of anti-platelet agents or statins were not considered. These could act as residual confoundings. Fourth, serum P concentrations vary by as much as 50% on a normal day, which reflects the effect of food intake but also an underlying circadian rhythm [37]. However, blood samples were drawn at morning after at least 8-hour fasting in this study, so a potential bias from wide physiologic variation could be minimized. Fifth, chronic inflammation, one of the important factors related with vascular calcification, was not considered. We did not have data for inflammatory markers such as C-reactive protein. Although our models included serum albumin level which can reflect inflammatory state in part, it might be insufficient. Last, a cross-sectional design precludes the evaluation of temporality and causality. In addition, CAC is an intermediate outcome. Although CAC is a good marker of future coronary events, the association found here should be carefully interpreted. Further studies using crucial clinical outcomes such as incident coronary artery disease or CV mortality are warranted.

In conclusion, a high serum P concentration, even within normal range, may be significantly associated with CAC in subjects without renal dysfunction. This association can be generalized beyond racial and regional difference. It may be important concern in the view of public health, and further studies are warranted indeed.

## Supporting Information

S1 Dataset The final dataset of this study (XLSX)
